# Ceramide synthase 2 deletion decreases the infectivity of HIV-1

**DOI:** 10.1016/j.jbc.2021.100340

**Published:** 2021-01-28

**Authors:** Eric Barklis, Ayna Alfadhli, Jennifer E. Kyle, Lisa M. Bramer, Kent J. Bloodsworth, Robin Lid Barklis, Hans C. Leier, R. Max Petty, Iris D. Zelnik, Thomas O. Metz, Anthony H. Futerman, Fikadu G. Tafesse

**Affiliations:** 1 Department of Molecular Microbiology and Immunology, Oregon Health and Sciences University, Portland, Oregon, USA; 2 Biological Sciences Division, Earth and Biological Sciences Directorate, Pacific Northwest National Laboratory (PNNL), Richland, Washington, USA; 3 Computing and Analytics Division, National Security Directorate PNNL, Richland, Washington, USA; 4 Department of Biomolecular Sciences, Weizmann Institute of Science, Rehovot, Israel

**Keywords:** ceramide, ceramide synthase, sphingolipid, HIV, fusion protein, β-gal, β-galactosidase, BME, β-mercaptoethanol, CA, capsid, CerS, ceramide synthase, CT, cytoplasmic tail, Env, envelope, G, glycoprotein, HEK293T, human embryonic kidney 293T, HexCer, hexosylceramide, NIH, National Institutes of Health, PIP2, phosphatidyl-4,5-bisphosphate, PM, plasma membrane, PrGag, precursor Gag, PS, phosphatidylserine, SL, sphingolipid, VSV, vesicular stomatitis virus

## Abstract

The lipid composition of HIV-1 virions is enriched in sphingomyelin (SM), but the roles that SM or other sphingolipids (SLs) might play in the HIV-1 replication pathway have not been elucidated. In human cells, SL levels are regulated by ceramide synthase (CerS) enzymes that produce ceramides, which can be converted to SMs, hexosylceramides, and other SLs. In many cell types, CerS2, which catalyzes the synthesis of very long chain ceramides, is the major CerS. We have examined how CerS2 deficiency affects the assembly and infectivity of HIV-1. As expected, we observed that very long chain ceramide, hexosylceramide, and SM were reduced in CerS2 knockout cells. CerS2 deficiency did not affect HIV-1 assembly or the incorporation of the HIV-1 envelope (Env) protein into virus particles, but it reduced the infectivites of viruses produced in the CerS2-deficient cells. The reduced viral infection levels were dependent on HIV-1 Env, since HIV-1 particles that were pseudotyped with the vesicular stomatitis virus glycoprotein did not exhibit reductions in infectivity. Moreover, cell–cell fusion assays demonstrated that the functional defect of HIV-1 Env in CerS2-deficient cells was independent of other viral proteins. Overall, our results indicate that the altered lipid composition of CerS2-deficient cells specifically inhibit the HIV-1 Env receptor binding and/or fusion processes.

As an enveloped virus, the HIV-1 depends on host cell lipids to assemble replicating viruses (1, 2). Among the lipids enriched in the envelopes of HIV-1 particles are phosphatidyl-4,5-bisphosphate (PIP2), phosphatidylinositol-3,4,5-trisphosphate, phosphatidylserine (PS), sphingomyelin (SM), and cholesterol (3, 4, 5, 6, 7, 8). Ample evidence has shown that binding of the matrix domains of HIV-1 precursor Gag (PrGag) proteins to PIP2 molecules directs virus assembly at the plasma membranes (PMs) of infected cells (9, 10). Investigations also have demonstrated that depletion of HIV-1 cholesterol levels reduces viral infectivity, and this effect can be mitigated in part by mutations in the cytoplasmic tail (CT) of the HIV-1 envelope (Env) protein (11, 12, 13, 14, 15, 16, 17). However, potential roles of other enriched viral lipids, including SM, have not been elucidated.

As illustrated in Figure 1, cellular levels of SM and other sphingolipids (SLs) are closely associated with the metabolism of ceramide (Cer; (18, 19, 20, 21)). Notably, major pathways for the production of Cer from sphingosine and sphinganine (dihydrosphingosine) are governed by the activities of different ceramide synthase (CerS) enzymes (18, 19, 20, 21). Mammals encode six CerS variants (CerS1–CerS6) that are differentially expressed in different tissues and have different chain length preferences (21, 22, 23, 24). In particular, CerS5 and CerS6 have short acyl chain specificities (C14 and C16), CerS1 and CerS4 have long chain specificities (C18 and C20), whereas CerS2 and CerS3 have very long chain specificities (C20–C30) (21).

**Figure 1:**
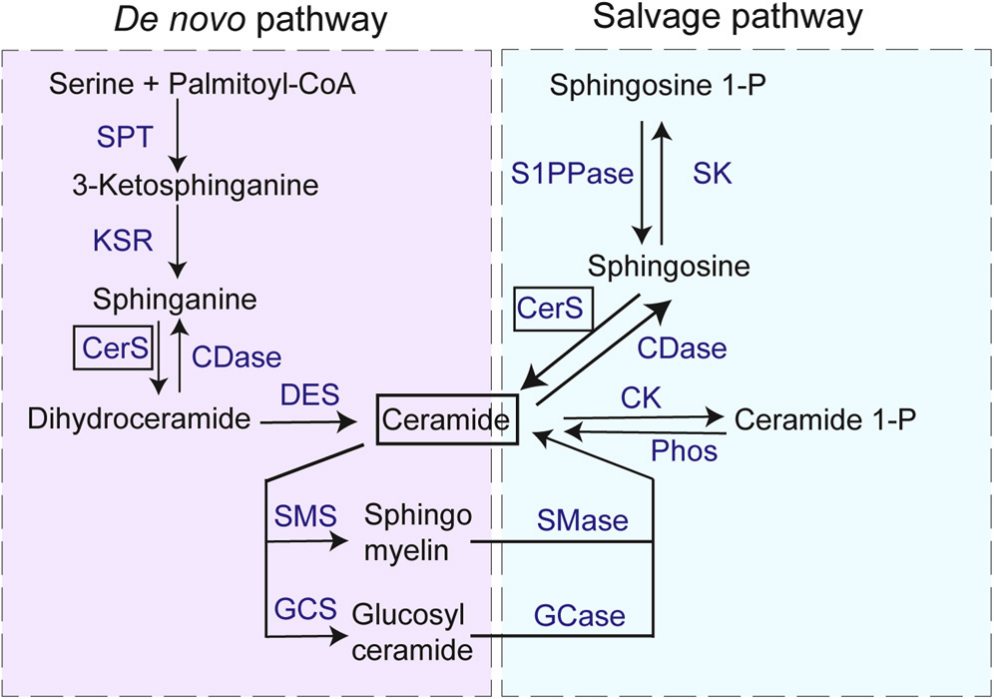
**Sphingolipid metabolism.** Shown are the pathways that regulate sphingolipid levels in mammalian cells. As illustrated, the *de novo* synthetic pathway uses serine palmitoyl-CoA-acyltransferase (SPT) to produce 3-ketosphinganine (3-keto-dihydrosphingosine), which is converted to sphinganine (dihydrosphingosine) through the action of 3-ketosphinganine reductase (KSR). Sphinganine is converted to ceramides *via* sequential reactions employing one of the ceramide synthases (CerS) and ceramide desaturase (DES). The salvage pathway involves the conversion of sphingosine 1-P to sphingosine by sphingosine-1-phosphate lyase (S1PPase) and the CerS-mediated production of ceramide from sphingosine. An alternate pathway for generation of ceramides is *via* phosphatase (Phos) action on ceramide 1-P, whereas ceramides and sphingomyelins are interconverted through the activities of sphingomyelinase (SMase) and sphingomyelin synthase (SMS). Ceramides and glucosylceramides also are interconverted, using the enzymes glucosylceramide synthase (GCS) and glucosyl ceramidase (GCase), and glucosylceramides are used as substrates for the synthesis of other hexosylceramides, as well as cerebrosides and gangliosides. Other enzymes involved in sphingolipid metabolism include ceramidase (CDase), ceramide kinase (CK), and sphingosine kinase (SK).

We have examined the effects of knocking out CerS2 on the assembly and infectivity of HIV-1. Lipid analyses showed that relative to WT cells, CerS2−/− cells had moderately reduced levels of long chain Cer species and greatly reduced levels of long chain SMs and hexosylceramide (HexCer) lipids. Importantly, while CerS2−/− cells supported the efficient assembly and release of viruses containing normal amounts of HIV-1 Env proteins, viruses so obtained were a third as infectious as those produced in WT cells. Similar results were obtained with HIV-1 virions carrying an Env protein cytoplasmic domain deletion (ΔCT; (17, 25, 26)), but infectivities of HIV-1 virions pseudotyped (27, 28) with the vesicular stomatitis virus (VSV) glycoprotein (G) were not so affected. Cell–cell fusion assay results mimicked virus infection results, indicating that the Env protein defect in CerS2−/− cells was independent of other virally encoded constituents. Overall, our results demonstrate that the HIV-1 Env binding and/or fusion functions are dependent on membrane SL compositions.

## Results

### Lipid analysis of CerS2−/− cells

To monitor the effects of CerS2 mutations on cellular lipid compositions, we performed lipidomic analyses on WT human embryonic kidney 293T (HEK293T) cells (29) and on HEK293T cells in which both CerS2 alleles were edited by CRISPR/Cas9 technology to possess premature stop codons after 63 residues (23). CerS2 is the predominant CerS expressed in the kidney (21, 30), and proteomic analyses of HEK293 cells have revealed that CerS2 is expressed at 10-fold higher levels than CerS5, whereas CerS1, CerS3, CerS4, and CerS6 were either not detected or detected at trace levels (31). Although some ceramides are present in the fetal bovine serum (32), and Cer can be generated by hydrolysis of SM or glucosylceramide (Fig. 1), the CerS-mediated pathways are major routes of SL production (18, 19, 20, 21). Because of this, we anticipated that CerS2−/− HEK293T cells might show reduced levels of Cer-derived SLs and, in particular, very long chain SLs.

**Figure 2:**
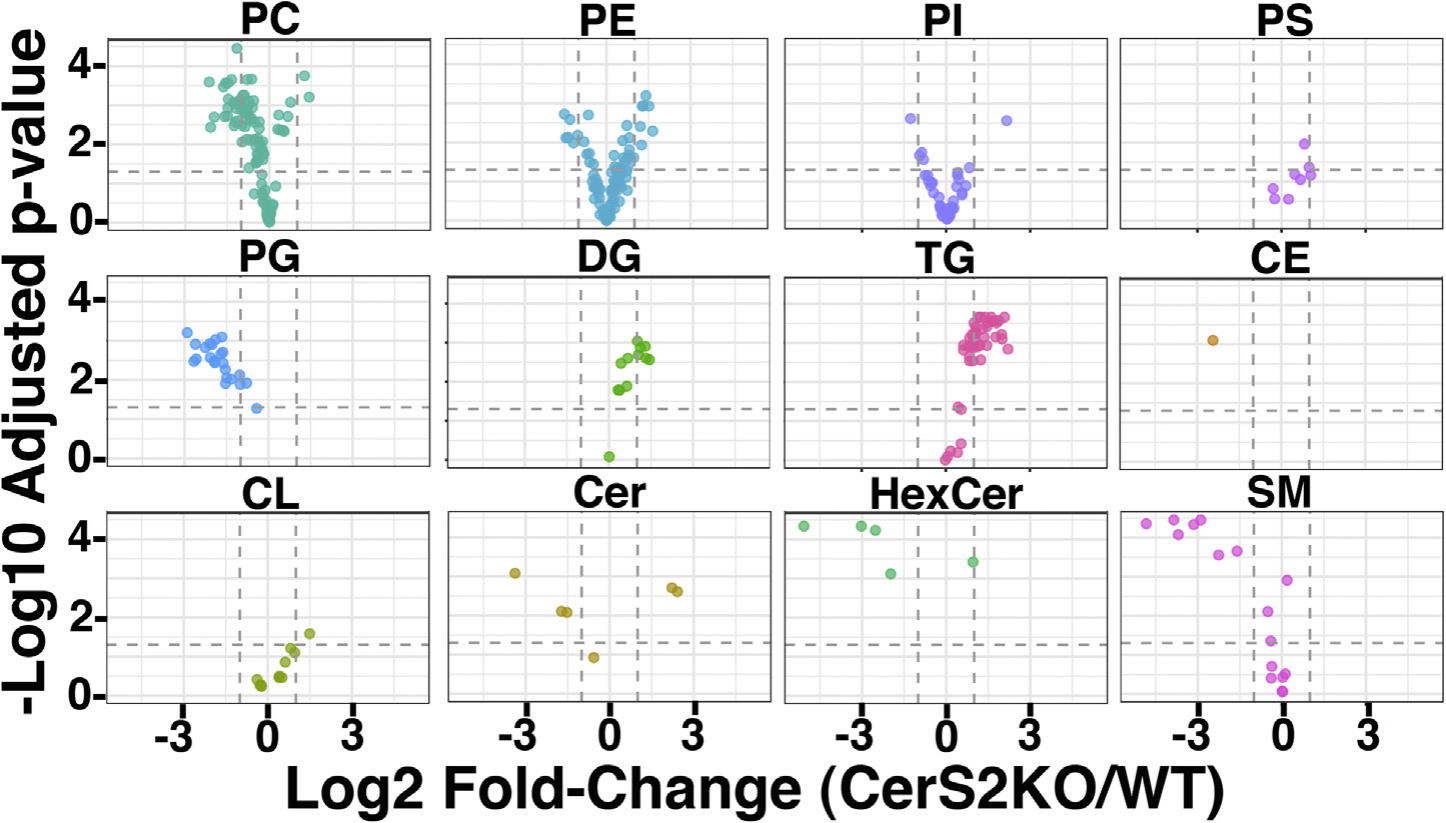
**Lipidomic analysis of CerS2−/− cells.** Lipidomic analyses of WT and CerS2−/− human embryonic kidney 293T cells were performed as described in the Experimental procedures section, and Volcano plots show comparisons of mutant cell lipids relative to WT cell lipids for the following lipid classes: phosphatidylcholine (PC), phosphatidylethanolamine (PE), phosphatidylinositol (PI), phosphatidylserine (PS), phosphatidylglycerol (PG), diacylglycerol (DG), triacylglycerol (TG), cholesteryl ester (CE), cardiolipin (CL), ceramide (Cer), hexosylceramide (HexCer), and sphingomyelin (SM). *X*-axes indicate log2 fold change of mutant/WT, *Y*-axes indicate −log10 adjusted *p* values, *horizontal dotted lines* correspond to a *p* value of 0.05, whereas *vertical dotted lines* correspond to a twofold change in means for the respective directions. As shown, DG and TG levels appeared slightly increased in CerS2−/− cells, and PC and PG levels appeared slightly reduced. Also, while levels of shorter chain ceramides (d18:1/16:0, d18:1/18:0, and d18:1/22:0) were increased or relatively unchanged in CerS2−/− cells, longer chain ceramides (d18:1/23:0, d18:1/24:0, and d18:1/24:1) were reduced. However, the greatest observed reductions were for very long chain hexosylceramides (d18:1/22:0, d18:1/23:0, d18:1/24:0, and d18:1/24:1) and sphingomyelins (d18:1/22:1, d18:1/24:0, d18:1/24:1 [or d18:2/24:0], d18:1/25:0, and d18:1/26:0). Complete analyses are provided in Table S1. CerS2, ceramide synthase 2.

For our analyses, lipids extracted from WT and CerS2−/− HEK293T cells were analyzed by LC/MS. In all, we identified 366 lipid species for which comparisons could be made (Table S1). These included phosphatidylcholines, phosphatidylethanolamines, phosphatidylinositols, PSs, phosphatidylglycerols, diacylglycerols, triacylglycerols, cholesterol ester, cardiolipins, Cers, SMs, and HexCers. Our results are visualized as volcano plots (Fig. 2), in which *X*-axes indicate log2 fold change of mutant/WT, and *Y*-axes indicate −log10 adjusted significance (*p*) values. Of the lipid classes, levels of phosphatidylcholines, phosphatidylethanolamines, phosphatidylinositols, PSs, and cardiolipins were only marginally affected in the CerS2−/− cells (Fig. 2). In contrast, diacylglycerols and triacylglycerols were slightly increased, and cholesterol ester and phosphatidylglycerols were slightly decreased. Interestingly, levels of shorter chain Cer species (d18:1/16:0 and d18:1/18:0) were increased in CerS2−/− cells, whereas the longest chain species (d18:1/23:0, d18:1/24:0, and d18:1/24:1) were decreased: these results are consistent with the role of CerS2 in very long chain Cer synthesis (21, 22, 23) and suggest a larger role for CerS5 in Cer production in mutant *versus* WT HEK293T cells. However, the most significant SL reductions in CerS2−/− cells were very long chain HexCer (d18:1/22:0, d18:1/23:0, d18:1/24:0, and d18:1/24:1) and SM (d18:1/22:1, d18:1/24:0, d18:1/24:1 [or d18:2/24:0], d18:1/25:0, and d18:1/26:0) species. Given that SMs are enriched in HIV-1 virions relative to host cell membranes (4, 6, 7, 8), we investigated how the lipid changes observed in CerS2−/− cells affected HIV-1 replication.

### CerS2−/− effects on HIV-1 replication

**Figure 3:**
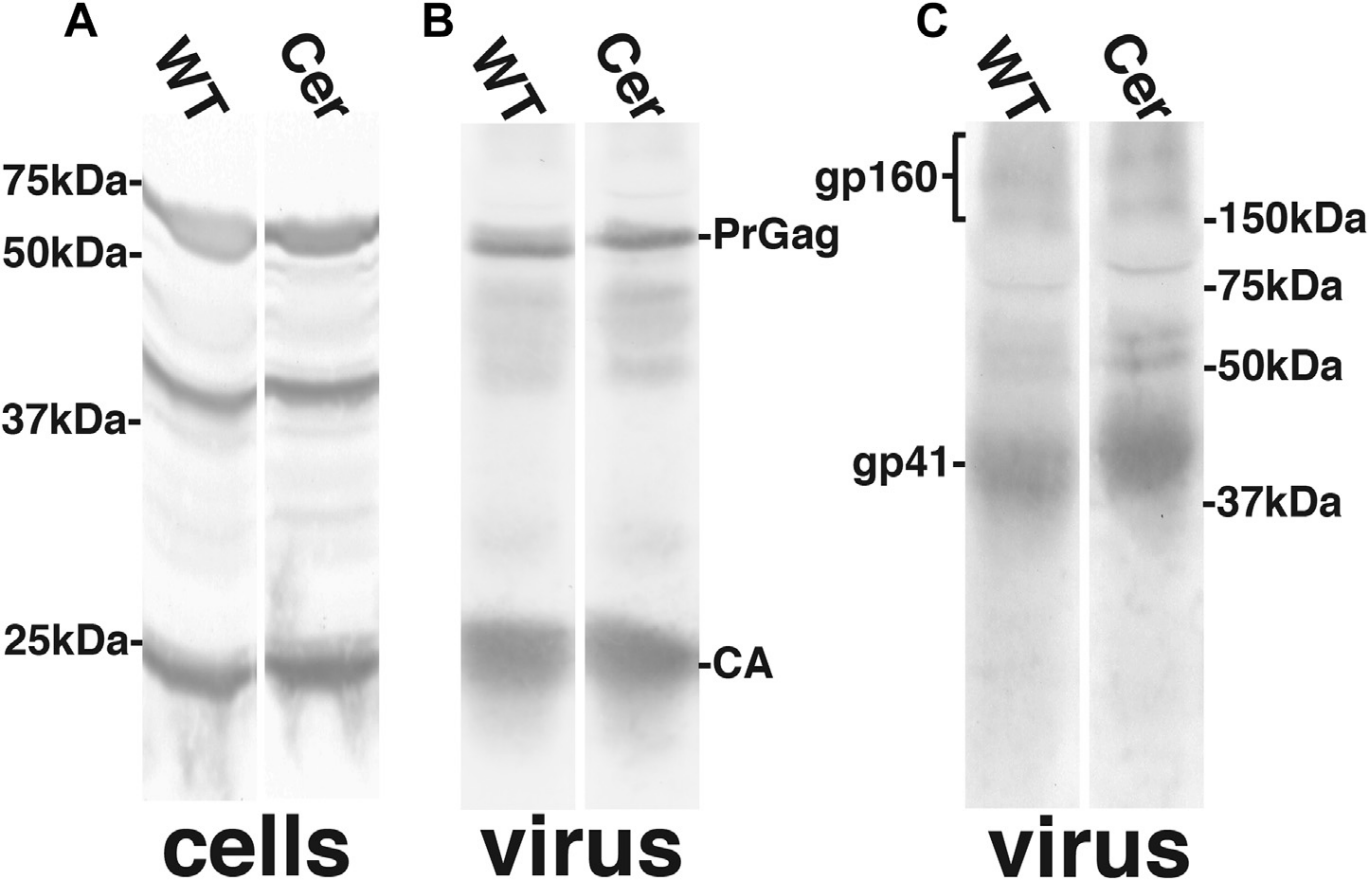
**HIV-1 virions released from WT and mutant cell lines.** WT and CerS2−/− human embryonic kidney 293T cells were transfected with the proviral HIV-1 NL4-3 construct. Three days post-transfection, cell lysate (cells) and virus samples were collected, and proteins were electrophoresed, and processed for detection of Gag proteins using an anti-CA antibody (*A* and *B*) and Env proteins using an anti-gp41 antibody (*C*). The migration positions gp41 and gp160 and of PrGag and CA are as indicated, and PrGag processing intermediates are also visible in the cell lysate samples. On the *far left* and *far right*, the positions of marker proteins run in parallel lanes are shown. CA, capside; CerS2, ceramide synthase 2; PrGag, precursor Gag.

To examine how a deficiency in CerS2 might affect HIV-1 assembly, release, and replication, we transfected WT and CerS2−/− HEK293T cells with the full-length NL4-3 (33) WT HIV-1 proviral plasmid and monitored particle production, Env levels in virus particles, and infection efficiencies. As shown in Figure 3*A*, cell lysate samples immunoblotted with an antibody against the Gag capsid (CA) protein showed similar staining patterns of PrGag, CA, and processing intermediates. Virus samples immunoblotted with the anti-CA antibody yielded the expected enrichment of CA over PrGag, with roughly equivalent levels of each species detected in samples produced from WT *versus* CerS2−/− cells (Fig. 3*B*). Virus samples immunoblotted with an antibody to the transmembrane (gp41) portion of Env also appeared similar (Fig. 3*C*), with appreciably higher levels of gp41 than the full-length unprocessed Env protein (gp160).

**Figure 4:**
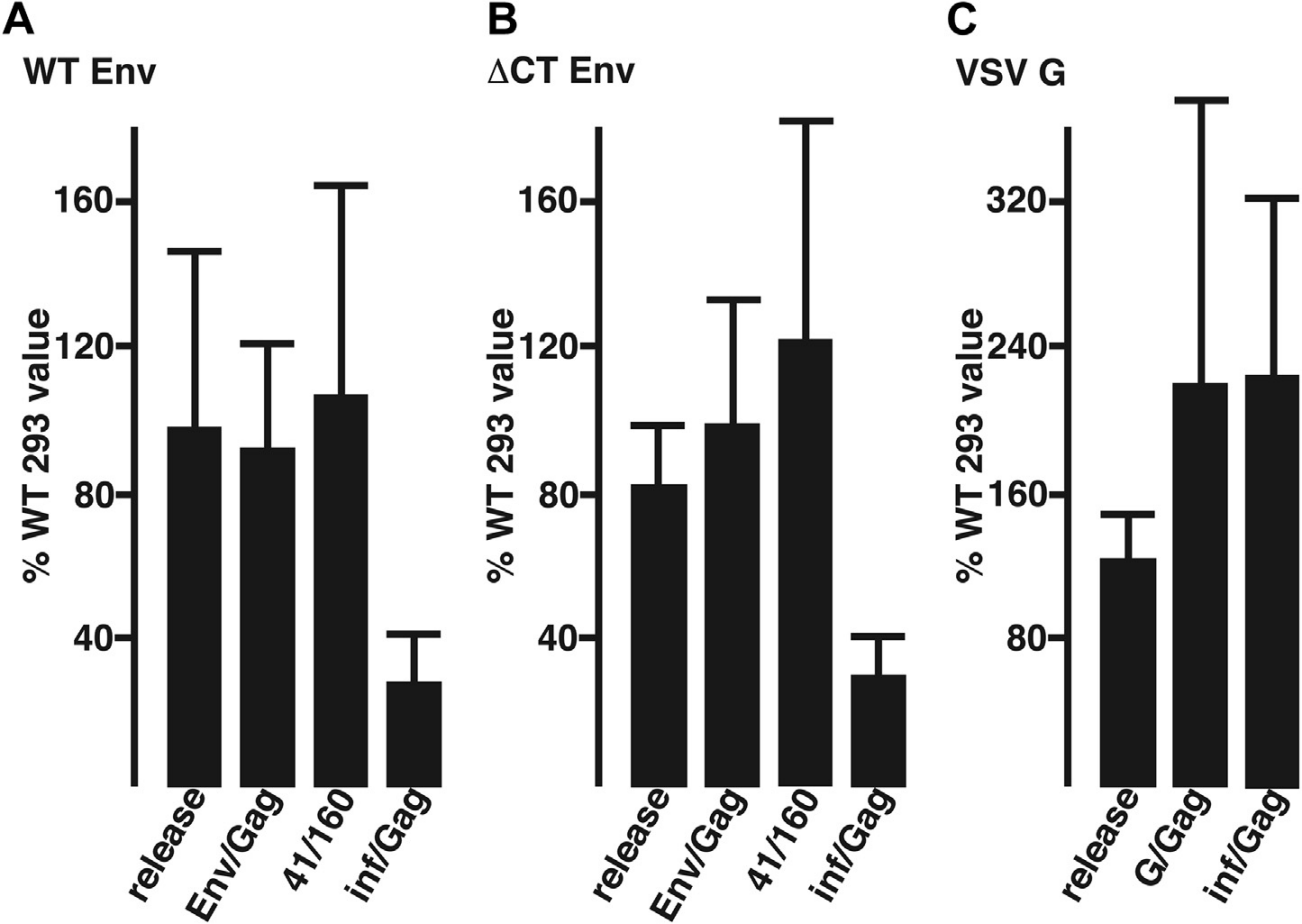
**Analysis of viruses released from WT and CerS2**−/− **cells.** WT and CerS2−/− human embryonic kidney 293T cells were transfected in parallel with constructs expressing WT HIV-1 (*A*), HIV-1 carrying an Env cytoplasmic domain deletion (ΔCT; *B*), or HIV-1 pseudotyped with the vesicular stomatitis virus glycoprotein (VSV G; *C*). Three days post-transfection, cell lysate and virus samples were collected, and processed for HIV-1 Gag, HIV-1 Env, and VSV G immunoblot detection to obtain viral release values (defined as viral Gag/cell Gag levels), viral Env/Gag ratios, and viral gp41/gp160 levels. Aliquots of viral samples also were used to infect TZM-bl reporter cells to quantify viral infectivities as described in the Experimental procedures section, and infectivity values were normalized to viral Gag levels to obtain inf/Gag ratios. In all panels, CerS2−/− values are depicted as percentages of WT human embryonic kidney 293T values. Results are averages and standard deviations from 10 independent experiments (*A*), six independent experiments (*B*), and five independent experiments (*C*). Note that WT HIV-1 and ΔCT HIV-1 release, Env/Gag, and gp41/gp160 ratios were not significantly different between the WT and mutant cell lines, but that mutant infectivity/Gag ratios were significantly reduced (*p* < 0.001) for viruses produced from mutant cells relative to WT cells. CerS2, ceramide synthase 2.

Quantitation of immunoblots from 10 independent pairwise transfections allowed us to compare WT and CerS2−/− HEK293T virus release levels (defined as viral *versus* cellular Gag levels), Gag-normalized Env levels in virions (Env/Gag), and ratios of gp41 to gp160 (41/160) in virions (Fig. 4*A*). As shown, no significant differences in these parameters were observed, indicating that CerS2 deficiency had little effect on HIV-1 assembly efficiencies, Env incorporation levels, or Env processing. To assay virus infectivities, viruses produced from WT and mutant cells were used to infect TZM-bl cells, which respond to HIV-1 infection, integration, and expression *via* the transactivation of their Tat-inducible β-galactosidase (β-gal) genes (34, 35, 36). Remarkably, we found a consistent 70% reduction in Gag-normalized infectivity (inf/Gag) levels from viruses produced in CerS2−/− cells *versus* WT cells (Fig. 4*A*).

The aforementioned results were reminiscent of previous studies, which demonstrated that perturbation of HIV-1 virion cholesterol levels reduced virus infectivities (11, 12, 13, 14, 15, 16, 17). Notably, HIV-1 resistance to cholesterol depletion has been mapped to the Env CT (13, 14, 15). Because of this, we also subjected an NL4-3 HIV-1 variant that carries a CT deletion (ΔCT) to our analyses. Although our first experiment suggested that ΔCT viruses might be slightly less sensitive than WT HIV-1 to CerS2 depletion, subsequent repeats showed that ΔCT viruses produced in CerS2−/− cells were as impaired for infectivity as WT viruses (Fig. 4*B*). We also tested two additional HIV-1 Env CT mutants: one of these was an Env P714L mutant (also called P203L (14, 15)) that was selected for resistance to cholesterol depletion; and the other was the Env 716Ins-R∗ mutant, which is cleaved by the HIV-1 protease (PR) in a similar fashion to P714L (26). These variants also showed significant infectivity reductions for viruses produced in mutant *versus* WT HEK293T cells (CerS2−/− P714L inf/Gag = 6% WT cells; CerS2−/− 716Ins-R∗ = 30 ± 11% WT cells). Overall, these results indicated that infectivity defects were independent of the Env CT.

The aforementioned data were consistent with an inhibition of receptor binding or virus–cell fusion or with subsequent steps of the HIV-1 replication cycle. As a means to narrow down possibilities, we performed our analyses with HIV-1 virions that were pseudotyped with the VSV G. Multiple studies have demonstrated that the VSV G protein can replace the function of HIV-1 Env and mediate the efficient binding, fusion, and entry of HIV-1 into target cells using alternate receptors and entry mechanisms (27, 28, 37, 38, 39). For our purposes, we pseudotyped Env-minus HIV-1 particles assembled by the HIV-Luc plasmid (40, 41) with VSV G and examined viruses produced in WT *versus* CerS2−/− HEK293T cells (see Experimental procedures section). Results from these pseudotype experiments are graphed in Figure 4*C*. As we observed with WT and ΔCT HIV-1, virus release of pseudotyped viruses was not impaired in mutant HEK293T cells. However, as opposed to WT and ΔCT viruses, Gag-normalized infectivities of pseudotyped viruses produced in CerS2−/− cells were more than twofold greater than the infection efficiencies of viruses from WT cells (Fig. 4*C*). We believe that this result can be accounted for by the observed increase in VSV G/Gag ratios in viruses released from the mutant cells (Fig. 4*C*). While we do not know why G proteins are assembled more efficiently into HIV-1 virions in CerS2−/− cells than WT cells, these results have specific implications. In particular, the observation that VSV G-pseudotyped viruses produced in mutant cells are not functionally impaired implies that the entry mechanism mediated by HIV-1 Env is perturbed in viruses generated from CerS2−/− cells.

### Cell–cell fusion assays

**Figure 5:**
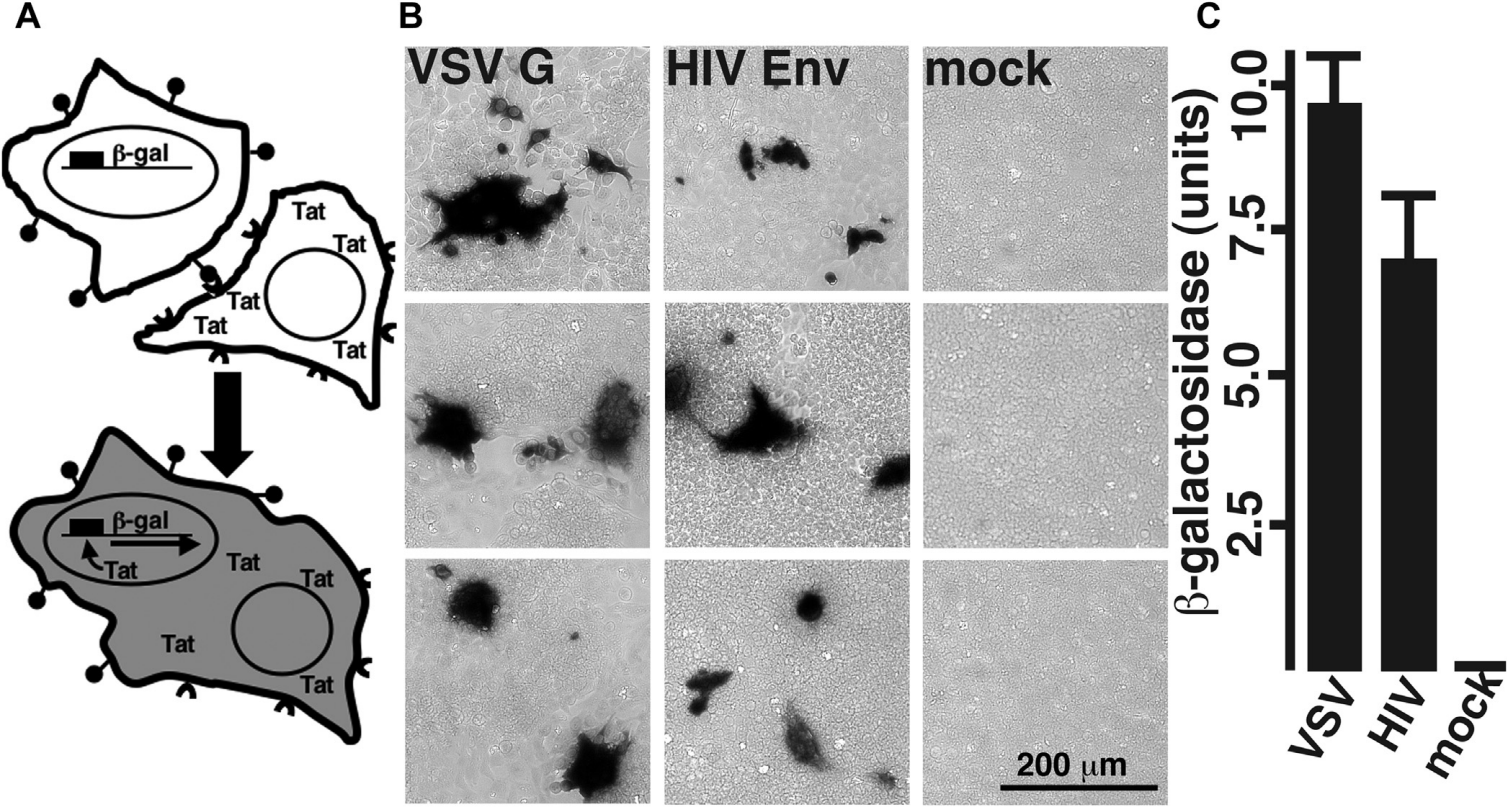
**Cell–cell fusion assays.** Cell–cell fusion assays were performed as depicted in panel *A*. As illustrated, TZM-bl cells, which express viral receptors (shown as *lollipops*) and a Tat-inducible β-gal gene, were coincubated with cells transfected to express viral envelope proteins (shown as *semicircles*) and Tat. Cell–cell fusion events result in the delivery of Tat to TZM-bl cells and the expression of β-gal. Fusion can be monitored as by X-gal staining for β-gal activity as shown in *B*, where TZM-Bl cells were incubated with cells transfected to express Tat plus either VSV G, HIV-1 Env, or nothing (mock). Here, fusion events were observed as black-staining clusters of cells. Note that the *middle* and *bottom* panels of the VSV G set of images contain overlapping elements that are rotated 90°, and that a 200 micron size bar for all images is shown in the bottom mock image. Fusion also can be quantified by incubation of cell lysates with colorimetric β-gal substrates such as ortho-nitrophenyl-β-galactoside (ONPG), as graphed from triplicate samples in panel *C*. Note that with VSV G, while some fusion activity was observed in the absence of low pH treatment, maximum fusion levels were observed 16 to 24 h after a 10 min (pH 5.5) treatment (see Experimental procedures section), consistent with a pH-triggered fusion mechanism. β-gal, β-galactosidase.

Given the results of our analyses of viruses produced from WT and CerS2−/− cells (Figs. 3 and 4), we reasoned that CerS2 depletion either exerted its effects directly on HIV-1 Env or on replication steps that are impacted by the different modes of entry mediated by HIV-1 Env and VSV G (27, 28, 37, 38, 39). To dissect these possibilities, we developed a cell–cell fusion assay. The approach we employed is cartooned in Figure 5*A*. As depicted, TZM-bl cells that harbor a Tat-inducible β-gal gene are incubated with cells that have been transfected to express the HIV-1 Tat protein along with a receptor binding and fusion protein such as HIV-1 Env or VSV G. On cell–cell fusion, Tat proteins activate the β-gal reporter. The *center* panel of Figure 5*B* illustrates the results of incubating TZM-bl cells with transfected cells expressing the Tat protein plus the indicated glycoprotein and staining for β-gal activity. As shown, no cells were stained in the absence of a receptor-binding and fusion protein (mock), but multiple stained syncytia were observed when VSV G or HIV-1 Env protein was coexpressed in transfected cells with Tat. In this regard, it is worth noting that while we observed some fusion activity with VSV G in the absence of any treatment, maximal VSV G-mediated fusion levels were elicited after a short low pH incubation (see Experimental procedures section), consistent with its pH-triggered fusion mechanism (37, 38). Figure 5*C* shows a quantitative assay of cell–cell fusion, in which β-gal activities were measured spectrophotometrically. Here cell–cell fusion events mediated by the VSV G and HIV-1 Env proteins yielded high levels of β-gal activity, whereas mocks gave background signals that were consistently more than 40 times lower.

**Figure 6:**
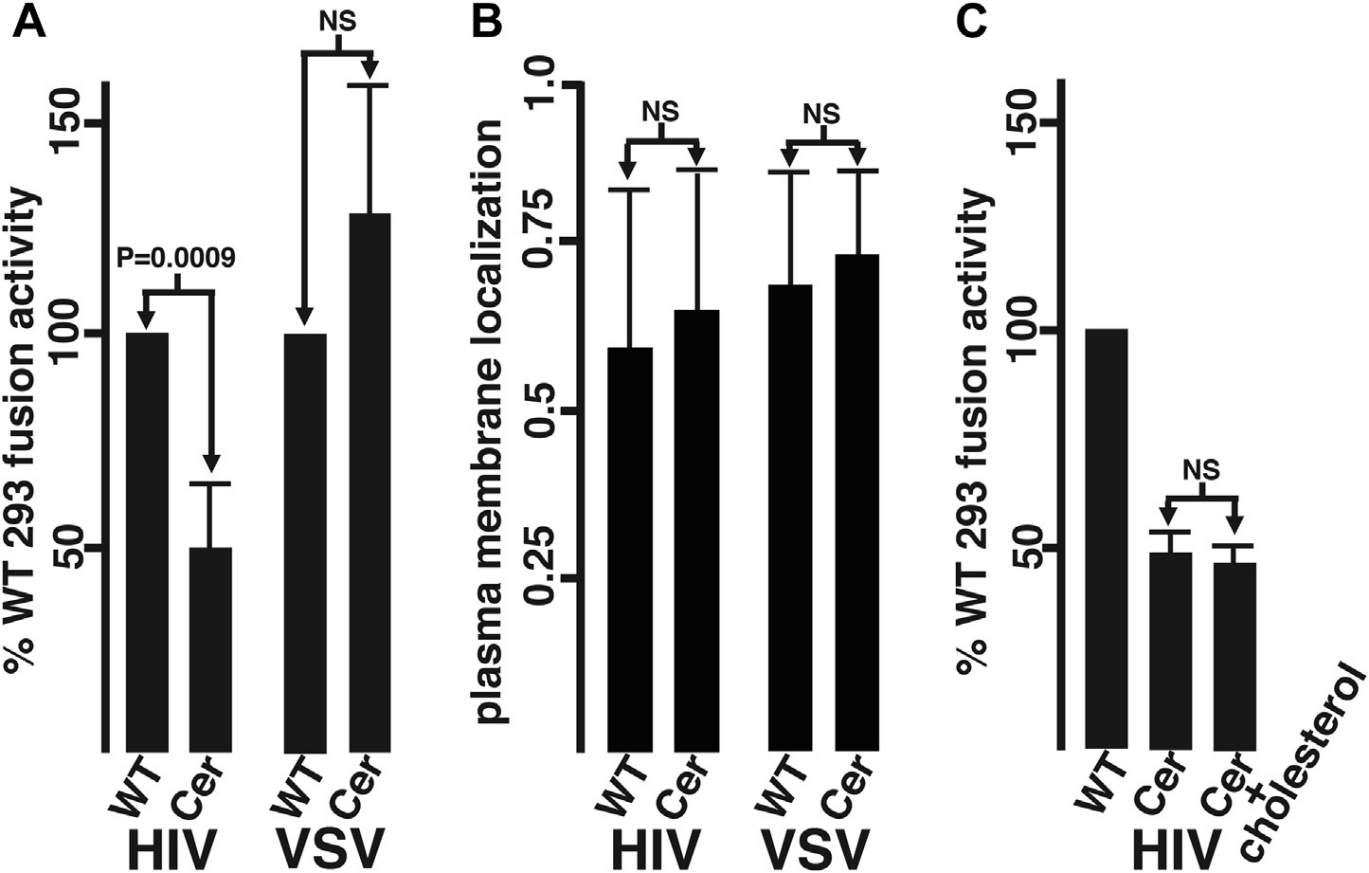
**CerS2 knockout effects on cell–cell fusion.***A*, WT and CerS2−/− human embryonic kidney 293T cells were transfected in parallel with expression vectors for either HIV-1 Env or VSV G, along with an expression vector for the HIV-1 Tat protein. At 24 h post-transfection, transfected cells were coincubated with TZM-Bl cells. At 48 h post-transfection, VSV G-transfected samples were subjected to a 10 min (pH 5.5) treatment (see Experimental procedures section), and β-gal assays were performed on all samples 16 to 24 h later. To control for transfection efficiencies, fusion activities were calculated by dividing β-gal levels by percentages of doubly positive Tat plus HIV-1 Env or VSV G cells, determined *via* immunofluorescence analysis of aliquots of transfected cells. Results are shown as fusion activities of CerS2−/− cells relative to the activities of WT cells performed in parallel and derive from four independent experiments. Averages and standard deviations are indicated. Note that the reduction observed for HIV-1 Env in CerS2−/− cells *versus* WT cells has a significance of *p* = 0.0009, whereas the slight increase for VSV G in CerS2−/− cells is not statistically significant (*p* = 0.19). *B*, to monitor plasma membrane localization of HIV-1 Env and VSV G in WT and CerS2−/− cells, cells were cotransfected with HIV-1 Env or VSV G expression vectors along with an expression vector for the PIP2-binding plasma membrane (PM) marker, PH-PLCδ1-GFP. Three days post-transfection, colocalization of PH-PLCδ1-GFP with Env or VSV G was determined microscopically as described in the Experimental procedures section. PM localization values were scored by calculation of Pearson's correlation coefficients for Env or VSV G *versus* PH-PLCδ1-GFP from at least 20 pairs of images. As shown, PM localization values appeared slightly higher for CerS2−/− cells than WT cells, but observed differences were not statistically significant. *C*, potential cholesterol effects were assessed by comparing cell–cell fusion values for WT cells, CerS2−/− cells, and CerS2−/− cells that were supplemented with cholesterol. Cholesterol assays were performed as detailed in the Experimental procedures section and indicated that cholesterol supplementation increased CerS2−/− cell cholesterol levels 3.4-fold. As shown, cholesterol supplementation yielded no statistically significant changes in CerS2−/− cell–cell fusion activities. CerS2, ceramide synthase 2; Env, envelope; VSV G, vesicular stomatitis virus glycoprotein.

Utilizing cell–cell fusion assays, we examined the fusion activities of the VSV G and WT HIV-1 Env proteins expressed in CerS2−/− and WT HEK293T cells following the protocol described in the Experimental procedures section. Our results mimicked our results with virus particles. Figure 6*A* graphs the observed fusion activities from four independent experiments of HIV-1 Env and VSV G expressed in mutant cells relative to WT cells. Importantly, the twofold reduction in HIV-1 Env fusion activity was calculated to be highly significant (*p* = 0.0009). The observed fusion defect of HIV-1 Env in CerS2−/− cells did not appear to be CT dependent, as fusion assays with the ΔCT HIV-1 Env variant also yielded reduced fusion activities for ΔCT Env expressed in mutant *versus* WT HEK293T cells (38%; data not shown). The fusion defect of HIV-1 Env in mutant cells also did not appear to be due to decreased levels of Env PM localization, since both HIV-1 Env and VSV G showed similar PM localization levels in WT *versus* CerS2−/− cells, as monitored by colocalization with the PIP2 biosensor (42), pH-PLCδ1-GFP (Fig. 6*B*). This result is consistent with the observation that Env/Gag levels in viruses produced from WT and CerS2−/− cells were approximately equivalent (Fig. 4).

Because our lipidomic studies indicated a several-fold cholesterol reduction in CerS2−/− *versus* WT HEK293T cells (Fig. 2), it was of interest to assess whether the cell–cell fusion defects were a consequence of cholesterol levels. To address this, CerS2−/− cells were grown in the presence of exogenous cholesterol, using a supplementation strategy employed for the growth of cholesterol-dependent cell lines (43, 44). Total cholesterol assays performed as described in the Experimental procedures section indicated that cholesterol treatment increased CerS2−/− cholesterol levels 3.4-fold (data not shown). Despite this, HIV-1 Env-mediated cell–cell fusion levels for cholesterol-treated CerS2−/− cells remained at less than half of the levels observed for WT cells (Fig. 6*C*). Taken together, our data suggest that at least part, if not all, of the replication defects of HIV-1 expressed in CerS2−/− cells stem from impaired HIV-1 Env binding and/or fusion activities, and that the binding/fusion defects are not dictated by variations in cholesterol levels.

**Figure 7:**
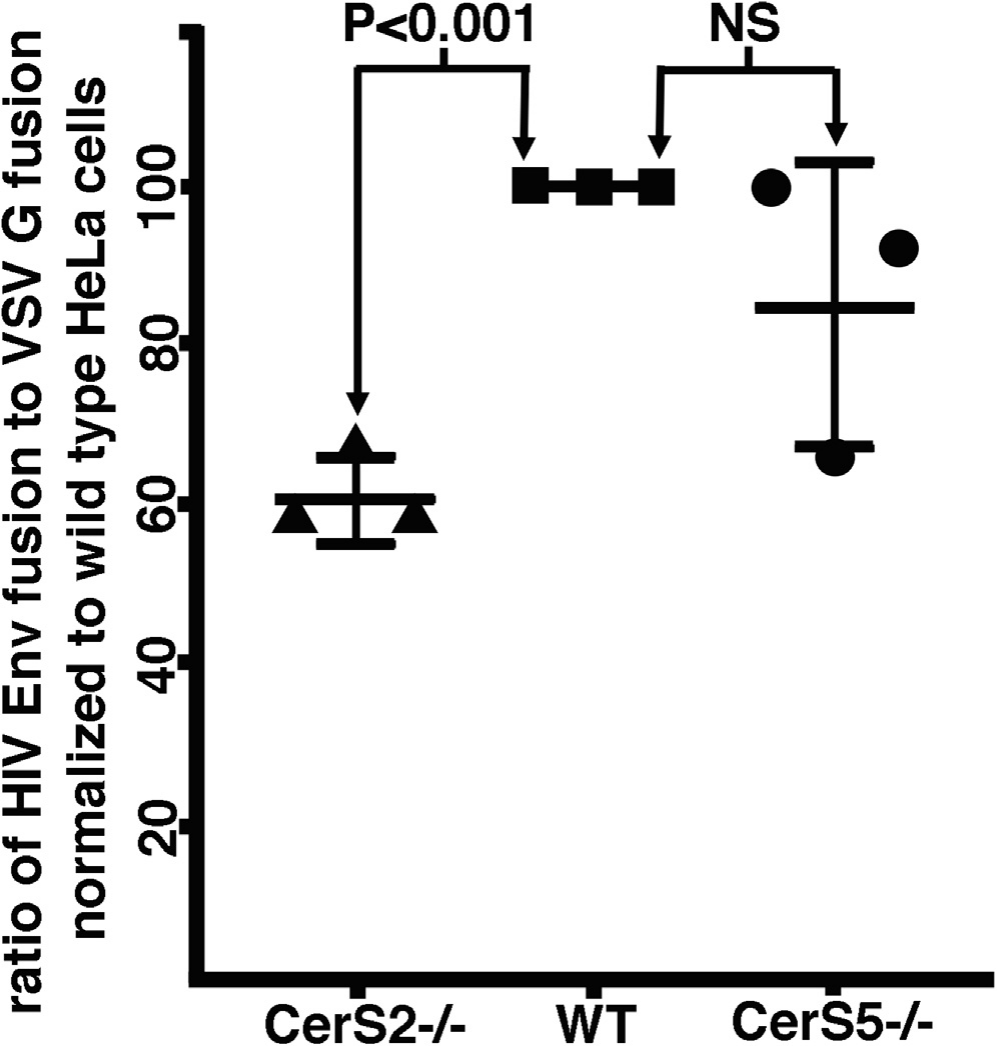
**Cell–cell fusion activities of HeLa CerS knockout cell lines.** WT HeLa cells (*squares*) and HeLa cells carrying knockouts of CerS2 (*triangles*) and CerS5 (*circles*) were cotransfected with expression vectors for VSV G or HIV-1 Env plus an expression vector for HIV-1 Tat and then were processed for cell–cell fusion assays as described for Figures 5 and 6. Results are graphed as the ratios of HIV-1 Env–mediated fusion levels *versus* VSV G–mediated fusion levels, normalized to WT HeLa cells, where a lower value indicates less efficient HIV-1 Env–mediated cell–cell fusion. Mean values and standard deviations are shown. The difference observed for the CerS2 knockout population *versus* WT HeLa has a significance of *p* < 0.001, whereas the difference for the CerS5 knockout *versus* WT HeLa was not significant (NS). CerS2, ceramide synthase; Env, envelope; VSV G, vesicular stomatitis virus glycoprotein.

To bolster our conclusions, we sought to determine whether our observations were specific to the CerS2−/− HEK293T cell line, or whether results might be similar in other CerS-deficient cells. To accomplish this, we undertook the analyses of a set of HeLa (42) cell–derived CerS knockouts that included WT HeLa cells, plus knockouts in CerS2 and CerS5, which are the major CerSs expressed in HeLa cells (31). These knockouts also were generated by CRISPR/Cas9 technology (24), but rather than clonal cell lines, they represented selected cell populations in which some, but not all cells, bore double allele knockouts. Nevertheless, recent lipidomic analyses indicated that the CerS5 knockout cells were reduced in short and long chain Cer and SM species, whereas CerS2 knockout cells were reduced in very long Cer, SM, and HexCer species (45). For our analyses, transfected HeLa cell variants expressing Tat plus either VSV G or HIV-1 Env were subjected to fusion assays with TZM-bl reporter cells as described in Figures 5 and 6. Our results (Fig. 7) are graphed as ratios of HIV Env fusion activities *versus* VSV G fusion activities, normalized to WT HeLa cells. As illustrated, we saw no statistical difference between WT HeLa cells and CerS5 knockout HeLa cells. In contrast, the fusion ratio for the HeLa CerS2−/− cells (61 ± 5%) was significantly less than WT HeLa levels (*p* < 0.001). This result is consistent with the observation that CerS2 is the predominant CerS expressed in HeLa cells (31) and supports the interpretation that the HIV-1 Env fusion defects we have characterized are not unique to the CerS2−/− HEK293T cell line.

## Discussion

Enveloped viruses are especially dependent on host cell lipids to fulfill their assembly and replication functions (2). HIV-1 exemplifies this dependence. Lipidomic analyses of HIV-1 particles have indicated that they are enriched in PIP2, phosphatidylinositol-3,4,5-trisphosphate, PS, SM, and cholesterol (3, 4, 5, 6, 7, 8). Previous studies have documented roles of PIP2 and cholesterol in HIV-1 assembly and replication (9, 10, 11, 12, 13, 14, 15, 16, 17), but less attention has been paid to the roles of SM and other SLs. To address this, we have taken advantage of the availability of CerS2−/− cells (23) to examine effects on HIV-1 assembly and replication. This is physiologically relevant because CerS2 is highly expressed in T cells, often along with CerS6 and, at lower levels, CerS5 (31, 46). Our lipidomic analyses of CerS2−/− HEK293T cells largely gave predicted results (Fig. 2 and Table S1). Notably, the longest chain Cer molecules were reduced in CerS2−/− cells, whereas shorter chain Cer derivatives were increased (Fig. 2 and Table S1), as previously observed in the livers of CerS2−/− mice (47). These observations are in keeping with the very long chain specificity of CerS2. In addition to reductions in very long chain ceramides, CerS2−/− HEK293T cells also exhibited dramatic reductions in longer chain SM and HexCer species (Fig. 2 and Table S1). These observations reflect the importance of CerS in the synthesis of SM and HexCer (Fig. 1).

Our data do not implicate CerS2 as affecting HIV-1 release, Env incorporation into virions, or Env processing (Figs. 3 and 4). However, our data clearly show that HIV-1 virions produced in CerS2-deficient cells are replication impaired (Fig. 4). The replication defect is not dependent on the Env CT (Fig. 4*B*) but does involve the Env protein, as VSV G pseudotyped HIV-1 virions were not defective (Fig. 4*C*). In the absence of any other data, these results suggest possible defects in Env receptor binding, fusion, or subsequent replication steps that might be affected by the different modes of entry directed by VSV G *versus* HIV-1 Env (1, 37, 38, 39). Our cell–cell fusion assays have helped narrow these options. In particular, the different cell–cell assay outcomes mediated by HIV-1 Env and VSV G (Fig. 6) support the following interpretations: at least part of replication defect of viruses produced in CerS2−/− cells is attributable to the binding and/or fusion function of HIV-1 Env; and the Env function defect is not dependent on other HIV-1 proteins.

Our cell–cell fusion assays also allowed us to demonstrate that the observed impairment of Env function was not an anomaly of HEK293T CerS−/− cells but also occurred in independently derived HeLa CerS−/− cell populations (Fig. 7). We also endeavored to test whether inhibitors of lipid metabolism might be used to confirm our results. Unfortunately, these efforts were hampered for a number of reasons. One issue is that removal of inhibitors from viral stocks requires centrifugation and resuspension steps that variably alter virus infectivities. We also saw that the presence of fumonisin B, and inhibitor of CerS (18, 19, 20), not only inhibited HIV-1 infections but also infections with VSV G-pseudotyped HIV-1 virions (data not shown), making it difficult to disentangle HIV-1–specific effects from potential pleiotropic (18, 19, 20) effects.

One of the questions arising from our studies concerns the identity of the specific lipid changes that have impacted HIV-1 replication. Because SM levels are enriched in HIV-1 particles while Cer levels are reduced (8), it is tempting to speculate that reductions of very long chain SMs in the outer leaflets of HIV-1 particles might impair the HIV-1 Env fusion process. However, evidence suggests that it is the PrGag protein that confers membrane selectivity (9, 10, 17, 48, 49). Thus, the pertinent lipid environment in cell–cell fusion assays, which exclude PrGag, is unknown. In this regard, it is worth mentioning a number of studies that have implicated SLs as modulators of HIV-1 infections (50, 51, 52, 53, 54). Several reports have documented the importance of viral gangliosides in the recognition of HIV-1 particles by dendritic cells (50), but these interactions appear unrelated to the HIV-1 Env binding and fusion process. More directly related to our studies are investigations that examined how alterations of target cell SL compositions have impacted HIV-1 infection and Env-mediated fusion (51, 52, 53, 54). Studies by Finnegan *et al.* (51, 52) demonstrated that pharmacologically or enzymatically induced increases in target cell Cer levels reduced HIV-1 infectivity at an Env-dependent postbinding fusion-related step. Related studies indicated that inhibition of glycosphingolipid synthesis in target cells also inhibited HIV-1 infection and Env-mediated fusion (53, 54). These observations clearly indicate that SL compositions in target cells can regulate HIV-1 Env function, but how they relate to our results, in which Env-embedded donor membranes were modulated, remains to be elucidated. In this regard, we believe that future detailed analyses of SL effects on Env trimer conformations (16, 55, 56) and fusigenic properties (57) may prove illuminating. We also believe that testing Env binding and fusion functions in direct virus–cell fusion assays would complement and extend our current analyses. It also might be worthwhile to examine the effects of CerS2 knockdown on the activities of other viral fusion proteins. Given that HIV-1 assembles at detergent-resistant lipid raft domains while VSV does not (11, 12, 13, 14, 15, 16, 17, 48, 49, 58, 59), it seems plausible that respective viral fusion proteins have adapted to operate most efficiently in ordered (HIV-1) *versus* fluid (VSV) membranes. This hypothesis is consistent with our results (Fig. 6), assuming that CerS2 depletion shifts the PM lipid environment of VSV G and HIV-1 Env from more ordered to more fluid. Thus, testing effects on the fusion functions of influenza virus *versus* Semliki forest virus, which respectively have ordered *versus* fluid membranes (58), would be of interest.

## Experimental procedures

### Cells

HEK293T cells (29) and HIV-1 reporter TZM-bl cells (34, 35, 36), respectively, were obtained from the American Type Culture Collection and the National Institutes of Health (NIH) AIDS Reagent Program. CerS2−/− HEK293T cells were generated using CRISPR/Cas9 technology as previously reported (23): both CerS2 alleles in these cells possess a premature stop codon after 63 residues (23). HeLa cells (60) also were obtained from the American Type Culture Collection, whereas pooled populations of HeLa CerS2 and CerS5 knockouts (kindly provided by Dr Howard Reizman) were generated using a CRISPR/Cas9 system based on the hypoxanthine phosphoribosyltransferase cotargeting approach as described previously (24, 61). The plasmid backbone for CerS single guide RNAs was pX330 (deposited by Feng Zhang, Broad Institute, as Addgene plasmid #42230), and the backbone for the hypoxanthine phosphoribosyltransferase 1 single guide RNA was pUC-U6-sg (61). Plasmids were cotransfected into HeLa cells using Lipofectamine 3000 (Thermo Fisher Scientific). At 5 days post-transfection, cells were selected with 6 μg/ml 6-thioguanine for 1 week, and populations so selected were employed in our analyses: lipid analyses of these cells have been described recently (45). All cells were grown in humidified 5% carbon dioxide air at 37 °C in Dulbecco's modified Eagle's medium supplemented with 10% fetal bovine serum plus 10 mM Hepes (pH 7.3), penicillin, and streptomycin.

### Virus analysis

WT HIV-1 virus stocks were generated by transfection of 10 cm plates of confluent cells with 24 μg of the HIV-1 proviral NL4-3 plasmid (33) using polyethyleneimine (26, 42), and collecting cell lysates and 0.45 micron-filtered viral supernatants at 72 h post-transfection as described previously (26, 36, 41, 42, 62). Mutant HIV-1 stocks similarly were prepared by transfection with NL4-3–derived Env ΔCT (CT144 (25, 26); obtained from Dr Eric Freed, NCI Frederick), Env P714L (P203L (14, 15), obtained from Dr Eric Freed, NCI Frederick), and Env 716Ins-R∗ (26) constructs. For pseudotyping of HIV-1 viruses with the VSV G, 10 cm plates of cells were transfected with 16 μg of HIV-Luc, an *env*-deleted HIV-1 expression plasmid (40, 41), plus 8 μg of the VSV G expression plasmid pMD.G (Addgene plasmid #12259; kindly provided by Dr Didier Trono).

Virus replication was measured in TZM-bl cells, following standard protocols (34, 35, 36). For each virus, triplicate samples of confluent TZM-bl cells in 6-well plates (diameter of 35 mm) each were incubated with 1 ml virus for 6 h, then supplemented with 1 ml media, and incubated for an additional 42 h. Following infection incubations, media were removed from cells, and cells from each well were scraped into 0.5 ml of PBS (9.5 mM sodium potassium phosphate [pH 7.4], 137 mM NaCl, and 2.7 mM KCl) and pelleted. Cell pellets were suspended in 150 μl PBS containing 0.1% SDS, vortexed, supplemented with 600 μl phosphate magnesium 2 buffer (;33 mM NaH_2_PO_4_, 66 mM Na_2_HPO_4_, 2 mM MgSO_4_, 0.1 mM MnCl_2_, 40 mM β-mercaptoethanol [BME]), vortexed, supplemented with 150 μl 4 mg/ml 2-nitrophenyl-β-d-galactopyranoside in phosphate magnesium 2 buffer, and incubated at 37 °C. Reactions were stopped by addition of 375 μl 1 M Na_2_CO_3_ and flash freezing on dry ice powder. Samples were then thawed, and light absorbances of 420 nm were read spectrophotometrically to calculate β-gal activities (1 unit = 1 nmol 2-nitrophenyl-β-d-galactopyranoside hydrolyzed per minute = 420 nm absorbance × 285/min of incubation; (63)) as a measure of infectivity (34, 35, 36).

To monitor Gag and Env levels in virus samples, aliquots of virus were concentrated by centrifugation through 2 ml 20% sucrose in PBS cushions (1 h at 197,000*g*; 40,000 rpm; Beckman SW41 rotor), suspended in 0.1 ml PBS, mixed with 0.1 ml of 2 × sample buffer (12.5 mM Tris–HCl [pH 6.8], 2% SDS, 20% glycerol, and 0.25% bromphenol blue) plus 0.1 volume of BME, and stored frozen prior to analysis. Samples were subjected to SDS-PAGE as described previously (26, 36, 41, 42, 63), except that samples for Env protein detection were not heated before electrophoresis, and acrylamide concentrations were either 10% or 7.5%. Typically, 15% viral samples were subjected to electrophoresis in parallel with molecular weight size standards (Bio-Rad). After SDS-PAGE fractionation, proteins were electroblotted and immunoblotted following previously described methods (26, 36, 40, 42, 62, 63). Primary antibodies employed were as follows: mouse anti-HIV-CA hybridoma media (Hy183; kindly provided by Dr Bruce Chesebro) at 1:15; mouse anti-gp41(CT) hybridoma media (Chessie 8 recognizing CT residues 727–732 [PDRPEG], from the NIH AIDS Reagent Program) at 1:15; human anti-gp41 (membrane proximal external region) (2F5; Polymun Scientific; Product AB001, Lot T580703-A, recognizing HIV-1 Env residues ELDKWA; kindly provided by Dr Hermann Katinger) at 1:15,000; and mouse anti-VSV G (Sigma–Aldrich; SAB4200695; clone P5D4) at 1:5000. Secondary antibodies (Promega) were antimouse or antihuman IgG alkaline phosphatase–conjugated antobodies used at 1:15,000. Color reactions for visualization of antibody-bound bands employed nitrobluetetrazolium plus 5-bromo-4-chloro-3-indolyl phosphate in alkaline phosphatase buffer (100 mM Tris–HCl [pH 9.5], 100 mM NaCl, and 5 mM MgCl_2_). For quantitation, immunoblots were air dried and scanned using an Epson Perfection V600 scanner. Band intensities of scanned tag image file format images were determined using NIH ImageJ software (64), and Env/Gag, gp41/gp160, and infectivity/Gag ratios were calculated from these intensities. Means and standard deviations from multiple experiments were converted to *Z* values assuming normal distributions, and statistical significance values were calculated from *Z* values.

Gag protein levels in cell lysate samples also were assayed to permit the monitoring of virus release. To do so, cell lysate samples were prepared by collecting cells in PBS, pelleting 20% of the cell sample, suspension in 50 μl IPB (20 mM Tris–HCl [pH 7.5], 150 mM NaCl, 1 mM EDTA, 0.1% SDS, 0.5% sodium deoxycholate, 1.0% TritonX-100, and 0.02% sodium azide), vortexing, incubation on ice for 5 min, pelleting 15 min at 13,000*g* to remove insoluble debris, mixing with 50 μl 2 × sample buffer plus 0.1 volume BME, and stored frozen prior to analysis as described for viral samples. Virus release levels were calculated as the ratios of viral Gag *versus* cellular Gag levels (41, 42, 62), and statistical significance values were calculated as described previously.

### Cell–cell fusion assays

Cell–cell fusion assays depended on the observation that fusion of cells expressing the HIV-1 Tat protein to TZM-bl cells resulted in the Tat-mediated activation of the HIV-1 promoter–driven β-gal gene (34, 35, 36). For standard assays, confluent 10 cm plates of cells were transfected as described previously with 12 μg of the Flag-tagged Tat expression vector pcDNA3.1-Tat101-Flag (from the NIH AIDS Reagent Program (65)) plus 12 μg of the WT HIV-1 Env expression vector (SVIIIEnvWT; SVIIIEnv, from the NIH AIDS Reagent Program (66, 67, 68)), a ΔCT Env expression vector (SVIIIEnvΔCT (68)), the pMD.G VSV G expression vector (62), or a control plasmid (Bluescript—SK (69)). Twenty-four hours post-transfection, cells were split onto coverslips for immunofluorescence analysis (see later) and 3 times 1:40 into 6-well plates. Six hours later, each well was supplemented with 500,000 TZM-bl cells, and the mixed cell cultures continued to grow at 37 °C in humidified 5% carbon dioxide air. At 48 h post-transfection, VSV G–transfected samples were subjected to a 10 min 37 °C treatment with serum-minus media that were adjusted to pH 5.5 with 10 mM Mes, after which they were refed with normal media and reincubated. β-gal assays were performed 16 to 24 h later as described previously, and in all experiments, cells transfected only with pcDNA3.1-Tat101-Flag plus a mock yielded β-gal signals that were at least 40 times lower than experimental sample signals.

To control for transfection efficiencies, transfected cells that were plated on coverslips were subjected to dual immunofluorescence analysis of Tat-Flag and HIV-1 Env or VSV G expression. Cells were fixed, permeabilized, washed, and subjected to successive rounds of primary and secondary antibody binding steps as described previously (42). For Tat-Flag detection, a mouse anti-Flag M2 antibody (Sigma F1804) was used at 1:5000; and an Alexa Fluor 488–conjugated antimouse secondary antibody (Invitrogen; Thermo Fisher) was used at 1:1000. For VSV G and HIV-1 Env detection, the primary antibodies noted previously were utilized at the indicated concentrations; and Alexa Fluor 594–conjugated antimouse or antihuman secondary antibodies (Invitrogen; Thermo Fisher) were employed at 1:1000. Samples were viewed on a Zeiss AxioObserver fluorescence microscope using 63× (Plan apochromat; numerical aperture = 1.4) objective and a Zeiss filter set 10 (excitation bandpass 450–490; beamsplitter Fourier transform 510; and emission bandpass 515–565) for green fluorophores, or Zeiss filter set 20 (excitation bandpass 546/12, beamsplitter Fourier transform 560, and emission bandpass 575–640) for red fluorophores. Images were collected with Zeiss Axiovision software, and percentages of doubly positive (Flag plus HIV-1 Env or Flag plus VSV G) cells were determined from collected images and used to obtain normalized fusion activity values. For cell–cell fusion experiments using HEK293T-derived cells, means and standard deviations from multiple experiments were converted to *Z* values assuming normal distributions, and statistical significance values were calculated from *Z* values. For experiments with HeLa-derived cells, values for the CerS2 knockout were compared with values from all other samples and were evaluated *via* one-way ANOVA and Tukey comparisons, using GraphPad Prism 5 software (GraphPad).

We also visualized cell–cell fusion incubations microscopically (Fig. 4). To do so, mixed cultures of transfected and TZM-bl cells were plated on coverslips. Following incubations, cells were fixed 15 min with 0.5% glutaraldehyde (Sigma–Aldrich) in PBS, rinsed with PBS, and incubated 4 to 24 h in PBS containing 5 mM K_4_Fe(CN)_6_, 5 mM K_3_Fe(CN)_6_, 1 mM MgSO_4_, 1 mg/ml 5'-bromo-4-chloro-3-indolyl-β-D-galactopyranoside (X-Gal; Roche 03117073001); and mounted on microscopic slides (42). Slides were viewed on a Zeiss AxioObserver fluorescence microscope using 63× (plan apochromat; numerical aperture = 1.4) objective under bright field illumination, and imaged using Zeiss Axiovision software.

### PM localization analysis

PM localization analyses of HIV-1 Env and VSV G proteins were performed by measuring colocalization with a PIP2-binding PM marker protein composed of the pleckstrin homology domain of PLCδ1 (PH-PLCδ1) fused to the GFP (42). To do so, WT and CerS2−/− HEK293T cells were transfected with expression vectors for either HIV-1 Env (SVIIIEnvWT) or VSV G (pMD.G) plus the PH-PLCδ1-GFP expression vector pLNCX2-PH-PLCδ1-GFP (42). Three days post-transfection, transfected cells on coverslips were processed for immunofluorescent detection of HIV-1 Env or VSV G and fluorescent detection of GFP as described in the cell–cell fusion assay given in Experimental procedures section. Images were collected with Zeiss Axiovision software with a fixed gain setting of 100, and exposures taken to maximize brightness levels without overexposure. Colocalization analysis was performed by determining Pearson's correlation coefficient values, which vary from −1 (inversely correlated) to +1 (completely correlated) (70). To do so, matched images in ImageJ (64) were stacked, single-cell areas without background regions were boxed and cropped, destacked, and then used as inupt for the ImageJ JACoP Pearson's correlation coefficient plugin (64). Colocalization values were averaged from at least 20 pairs of images for each sample. Values were evaluated *via* one-way ANOVA with Tukey comparisons, using GraphPad Prism 5 software (42).

### Lipid analysis

Lipids from WT and CerS2−/− HEK293T cells were analyzed utilizing a label-free and relative quantitation approach that has been employed in multiple previous studies (71, 72, 73, 74, 75, 76, 77, 78, 79). To do so, cells were washed 3 times with ice-cold PBS, detached from plates by scraping, transferred into glass sample tubes on ice, and centrifuged for 5 min at 500*g* at 4 °C. After aspiration of the PBS, cell pellets were resuspended in 1 ml of ice-cold methanol and stored at −80 °C. Using a modified Folch extraction (80, 81), chloroform and water were added to samples for a final ratio of 8:4:3 chloroform:methanol:water. The samples were vortexed to mix, chilled on ice for 5 min, and then vortexed again. The samples were incubated at 4 °C for 2 h to allow for the separation of the phases. The lower organic lipid–containing layer was removed, dried *in vacuo*, and then stored at −20 °C in 2:1 chloroform:methanol (v/v) until analysis.

For LC–MS/MS analysis and lipid identification, LC–MS/MS parameters and identifications were conducted as outlined (82). A Waters Aquity UPLC H class system interfaced with a Velos-ETD Orbitrap mass spectrometer was used for LC–electrospray ionization–MS/MS analyses. Lipid extracts were dried *in vacuo*, reconstituted in 10 μl of chloroform plus 540 μl of methanol, and injected onto a reverse-phase Waters CSH column (3.0 × 150 mm × 1.7 μm particle size), and lipids were separated over a 34-min gradient (mobile phase A: acetonitrile/H_2_O [40:60] containing 10 mM ammonium acetate; mobile phase B: acetonitrile/isopropanol [10:90] containing 10 mM ammonium acetate) at a flow rate of 250 μl/min. Samples were analyzed in both positive and negative ionization modes using higher-energy collision dissociation and collision-induced dissociation to obtain high coverage of the lipidome. Analysis of lipid abundance box plots confirmed that there were no notable differences between the extracted lipid samples in our analyses (Fig. S1). The fragment ions used for lipid identifications were used as previously outlined (82). The LC–MS/MS raw data files were analyzed using LIQUID software (82) whereupon all identifications were manually validated by examining the fragmentation spectra for diagnostic and fragment ions corresponding to the acyl chains. In addition, the precursor mass isotopic profile and mass ppm error, extracted ion chromatograph, and retention time for each identification were examined. To facilitate quantification of lipids, a reference database for lipids identified from the MS/MS data was created using LIQUID (82), and features from each analysis were then aligned to the reference database based on their identification, *m*/*z*, and retention time using MZmine 2 (83). Aligned features were manually verified, and peak apex intensity values were exported for statistical analysis.

Data from positive and negative ion modes were analyzed separately using MATLAB, version R2016b (MathWorks). Any unobserved lipid values were recorded as missing (not available), and the data were log2 transformed. The rMd-PAV algorithm (84) was used to identify potential outliers on the basis of their correlation, median absolute deviation, and skew; confirmation of outlier biological samples was achieved *via* Pearson correlation between the samples. All lipids were assessed for having at least two observations across all samples and enough observations for performing either qualitative or quantitative statistical tests (84); none of them failed to meet these requirements, and thus all lipids were retained for further analysis. The data were normalized using global median centering, in which each sample was scaled by the median of its observed abundance values. This approach has been described previously (85, 86, 87, 88) and utilized in a number of other studies (89, 90, 91). Lipids were evaluated using ANOVA with a Dunnett test correction to compare CerS2−/− *versus* WT samples. Yellowbrick (https://zenodo.org/record/1206264#.X2lTiBNKiMl) was used to perform recursive feature extraction.

It is notable that included among the SLs identified were several with odd chained fatty acids (Table S1). In this regard, although odd chain SLs are commonly present in low abundance, they have been identified by multiple laboratories in multiple cell types (89, 92, 93). Our identifications were performed using the LIQUID identification software (82) and confirmed *via* analysis of multiple fragment ions, the precursor mass isotopic profiles, extracted ion chromatograms, ppm error, and retention times of peaks.

### Cholesterol supplementation

To examine the effects of cholesterol supplementation on CerS2−/− HEK293T cells, cells were supplemented with cholesterol following protocols employed for the growth of cholesterol-dependent cell lines (43, 44). To do so, cell growth medium was supplemented with 0.1 mg/ml cholesterol (Sigma C8667) from a 10 mg/ml stock in ethanol, and cell–cell fusion assays were performed in the presence of added cholesterol. For determination of total cell cholesterol levels in the absence and presence of cholesterol supplementation, the Promega cholesterol Ester-Glo assay was employed, according to manufacturer's instructions. Briefly, 400,000 cells in 6-well plates were grown 2 days in the absence or the presence of 0.1 mg/ml cholesterol. Cells were collected in 1 ml PBS, washed five times with 1 ml PBS each wash, and suspended in 0.2 ml PBS. Aliquots of 2.5 μl were employed for total protein determinations using the Bio-Rad (catalog no. 500-0006) version of the Bradford (94) assay and bovine serum albumin (Sigma) as a standard. In parallel, 10 μl sample aliquots were mixed with 50 μl Promega cholesterol lysis solution, incubated 30 min at 37 °C, and transferred to a white-walled 96-well plate along with cholesterol standards. Wells were supplemented with 50 μl cholesterol detection reagent containing cholesterol esterase, cholesterol dehydrogenase, reductase, and proluciferin, and incubated 60 min at room temperature, prior to luminescence detection on a BMG Labtech CLARIOstar luminometer. Measurements indicated that the cholesterol supplementation regimen yielded a 3.4-fold increase in CerS2−/− HEK293T cholesterol levels.

## Data availability

All data are contained in the article plus Fig. S1 and Table S1.

## Conflict of interest

The authors declare that they have no conflicts of interest with the contents of this article.
