# Supplementary material for: Ceramide synthase 2 deletion decreases the infectivity of HIV-1
Source: J Biol Chem. 2021 Jan 28;296:100340. doi: 10.1016/j.jbc.2021.100340 (PMC7949126; doi:10.1016/j.jbc.2021.100340)
Supplement: Supplemental Figure S1 [file mmc2.pdf]

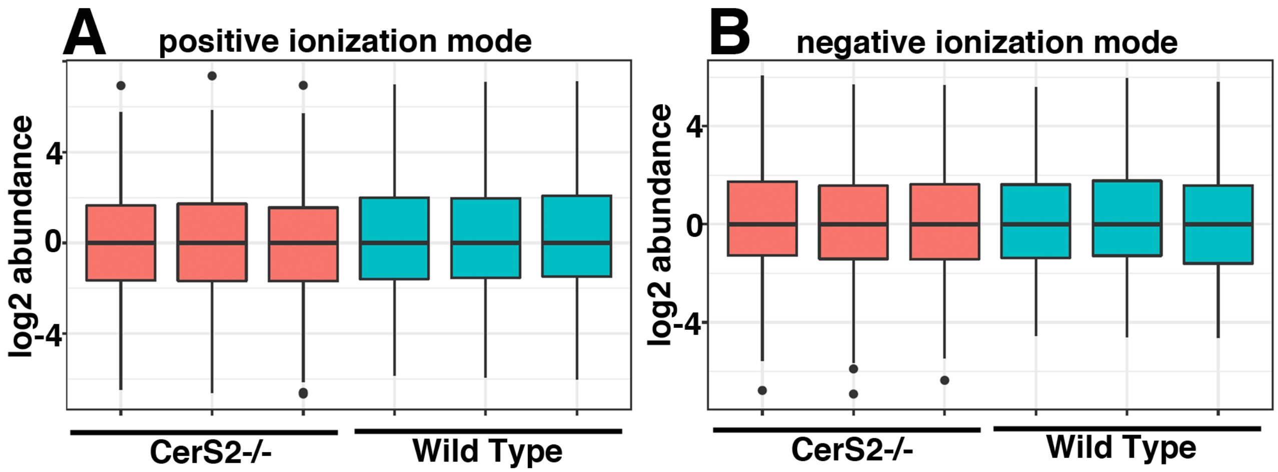

Supplementary Figure 1. Quality control and pre-processing of lipidomic data sets. Shown are standard boxplots of the full mass spectrometry datasets for each of the replicates in positive **(A)** and negative **(B)** modes. Boxplots were generated in R, version 3.6.0, and display the 25th and 75th percentiles (Q1 and Q3) as ends of the box. Whiskers extend to the maximum and minimum observed values, except in cases where observations were further than 1.5x(Q3-Q1) from the median, and those observations are indicated by points. Note that these data indicate no significant differences between samples.
